# EFFICACY OF PULMONARY REHABILITATION IN PATIENTS WITH CHRONIC OBSTRUCTIVE PULMONARY DISEASE AND OBSTRUCTIVE SLEEP APNOEA: A RANDOMIZED CONTROLLED TRIAL

**DOI:** 10.2340/jrm.v56.23757

**Published:** 2024-09-24

**Authors:** Honghua SHEN, Yiming XU, Yin ZHANG, Lei REN, Rui CHEN

**Affiliations:** 1 Department of Respiratory Rehabilitation, the Fourth Rehabilitation Hospital of Shanghai, Shanghai, China; 2 Department of Respiratory and Critical Care Medicine, Sleep Center, the Second Affiliated Hospital of Soochow University, Suzhou, China

**Keywords:** pulmonary rehabilitation, moderate-intensity aerobic exercise, positive airway pressure, COPD-OSA overlap syndrome

## Abstract

**Objective:**

To evaluate the efficacy of pulmonary rehabilitation in the outcomes of patients with chronic obstructive pulmonary disease–obstructive sleep apnoea overlap syndrome patients who used positive airway pressure.

**Design:**

Prospective randomized controlled single-blind trial.

**Patients:**

A total of 79 patients with chronic obstructive pulmonary disease–obstructive sleep apnoea overlap syndrome were randomly assigned to either the intervention group (*n* = 40) or control group (*n* = 39).

**Methods:**

All patients consistently adhered to positive airway pressure therapy every night from enrolment in the study, while intervention group patients received additional moderate-intensity aerobic exercise for 20 weeks. Pre- and post-intervention measurements included the 6-Minute Walk Test, Barthel Index, body mass index, fat mass, free fat mass, forced expiratory volume in 1 s (FEV1), FEV1%predicted, modified Medical Research Council, and polysomnography parameters.

**Results:**

After 20 weeks, the intervention group exhibited statistically significant improvements in 6MWD, Barthel Index, body mass index, fat mass, and modified Medical Research Council compared with control group (all *p* < 0.01). In addition, the intervention group showed a significantly lower percentage of total sleep time with oxygen saturation < 90% (*p* = 0.013) and higher lowest nocturnal oxygen saturation (*p* = 0.008) than the control group. However, there was no significant difference in FEV1 %predicted between the 2 groups.

**Conclusion:**

Pulmonary rehabilitation incorporating moderate-intensity aerobic exercise could improve physical endurance and motor abilities in individuals with chronic obstructive pulmonary disease–obstructive sleep apnoea overlap syndrome, while also improving anamnestic dyspnoea, body composition, and sleep-disordered breathing.

## LAY ABSTRACT

Chronic obstructive pulmonary disease and obstructive sleep apnoea are highly prevalent conditions, and the identification of coexisting obstructive sleep apnoea in chronic obstructive pulmonary disease patients holds significant clinical relevance. The presence of the overlap syndrome between chronic obstructive pulmonary disease and obstructive sleep apnoea can significantly contribute to a decline in physical endurance and impose limitations on motor abilities, thereby contributing to adverse health outcomes. The investigation of how appropriate pulmonary rehabilitation can enhance daytime physical endurance, nocturnal sleep hypoxia, and motor abilities should be thoroughly explored to improve the management of patients with chronic obstructive pulmonary disease and obstructive sleep apnoea overlap syndrome. This study employed moderate-intensity aerobic exercise to evaluate its efficacy in this patient population. The study demonstrated that incorporating additional moderate-intensity aerobic exercise alongside positive airway pressure therapy could effectively enhance physical endurance and ameliorate sleep-disordered breathing in patients diagnosed with the overlap syndrome of chronic obstructive pulmonary disease and obstructive sleep apnoea.

Chronic obstructive pulmonary disease (COPD) and obstructive sleep apnoea (OSA) are highly prevalent diseases affecting 10% and 5%, respectively, of the adult population > 40 years old, and their coexistence, which is defined as overlap syndrome, might occur in about 0.5% of this population (1–3). The greater than expected sleep-disordered breathing could be a major contributory factor to morbidity and mortality. The recognition of coexisting OSA in patients with COPD is clinically relevant, as the management of patients with overlap syndrome is different from the management of OSA or COPD alone. Continuous positive airway pressure (CPAP) is the first choice of treatment for patients with OSA as it reduces nocturnal respiratory events, symptoms, and cardiovascular morbidity (4, 5).

Physical inactivity could be a major burden for COPD-OSA overlap syndrome patients, as it is associated with poor health outcomes (6). Many patients experience exercise limitation (7, 8). Appropriate rehabilitation training can improve their quality of life (9). Pulmonary rehabilitation (PR) programmes should consider including a sleep assessment in patients with moderate to severe COPD and interventions when indicated to reduce the impact of OSA in COPD (10). However, for patients with COPD-OSA overlap syndrome, how to carry out effective PR and improve their daytime physical endurance, night sleep hypoxia, and motor ability should be investigated in depth.

The objective of this study was to evaluate the efficacy of a 20-week programme of PR with moderate-intensity aerobic exercise on physical endurance, motor abilities, body composition, PSG parameters, and respiratory function in patients diagnosed with COPD-OSA overlap syndrome who used positive airway pressure therapy.

## MATERIALS AND METHODS

### Study design and population

This prospective, randomized, controlled, single-blind trial with parallel group design was conducted at two medical centres, the Second Affiliated Hospital of Soochow University and the Fourth Rehabilitation Hospital of Shanghai, China, from January 2021 to August 2022, according to the principles of good clinical practice.

This study was approved by the Ethics Committee of the Second Affiliated Hospital of Soochow University (JD-LK-2018-004-02) and the Fourth Rehabilitation Hospital of Shanghai (SP202002). It was registered in www.chictr.org.cn/index.aspx (Trial registration: ChiCTR1800020257) prior to the recruitment of participants. All patients provided written informed consent, and the institutional human research ethics committee approved the study.

Patients suffering from both COPD and OSA were enrolled in the study according to the following criteria: (*i*) all patients with COPD were diagnosed based on the clinical history, physical examination, chest radiograph, and pulmonary function tests in accordance with the Global Initiative for Chronic Obstructive Lung Disease (GOLD) clinical criteria for the diagnosis and severity of COPD (9), and were inpatients or outpatients with stable COPD and no oxygen intake with peripheral oxygen saturation (SpO_2_) ≥88% (9); (*ii*) free from acute exacerbations for at least 4 weeks and reported habitual snoring during the clinical interview, and were patients who can complete all-night polysomnographic (PSG) and newly diagnosed with OSA; (*iii*) absence of any significant respiratory, neuromuscular, or other disorder that accounts for hypercapnia.

The exclusion criteria were as follows: (*i*) patients with wound-healing disorders, cognitive and/or mental disorders, linguistic deficits, exercise-limiting comorbidities, derangements in gas exchange and restrictive disease were excluded; (*ii*) concurrent treatment with anxiolytics or analeptic drugs and other associated chronic respiratory diseases; (*iii*) patients with overlap syndrome who started the treatment with PAP but showed adherence of < 3 h per night.

Patients provided informed consent before participation in the study. The protocols were based on international ethical standards.

### Randomization and blinding

Patients who fulfilled the inclusion criteria were randomized (allocation ratio 1:1) into the intervention group (IG) or control group (CG) using a computerized list. Also, the allocation was concealed in this trial. All assessments were conducted in a blinded manner by certified staff members not involved in clinical care (2 medical doctors with specialization in respiratory medicine, 3 physiotherapists, and 2 nurses).

Participants who met the inclusion criteria were randomized.

### Intervention

Patients in IG received positive airway pressure every night and participated in pulmonary rehabilitation during the daytime. CG patients received positive airway pressure only. The pulmonary rehabilitation programme consists of moderate-intensity aerobic exercise, conducted 5 times per week, with each session comprising a total of 40 min of exercise. Both cohorts of patients receive the prescribed intervention for a duration of 20 weeks.

### Outcomes

The 2 assessment time points were baseline and post-intervention. The following variables were collected at baseline: age, gender, weight, height, body mass index (BMI), smoking status, comorbidities, medication, arterial blood gases. Physical endurance was measured using the 6-Minute Walk Test (6MWT) and motor disability during basal activity of daily living by Barthel index (BI). Also, the number of COPD exacerbations requiring an emergency room visit, hospitalization, or outpatient visit was obtained.

### Six-minute walk test

The 6MWT was conducted following the American Thoracic Society (ATS)/European Respiratory Society (ERS) guidelines (11) to assess walking capacity on a 25-m linear pathway. Patients were instructed to cover maximum distance in 6 min on a level surface. The primary outcome measure was recorded walking distance in metres. Standardized instructions and motivational support were provided during the test. The 6MWT was conducted both pro- and post-PR programme for all participants.

### Pulmonary function tests

The spirometry tests were conducted by trained personnel in a dedicated, noise-free environment using the Master Screen device (Care Fusion Germany 234 GmbH, Hamburg, Germany). Each test was performed 3 times, and the optimal outcome from each test was recorded and utilized to derive the forced vital capacity (FVC), forced expiratory volume in 1 s (FEV_1_), and FEV_1_/FVC ratio. The modified British Medical Research Council (mMRC) questionnaire was employed to assess the severity of dyspnoea during daily activities.

### PSG study

All participants underwent a single night of PSG study, lasting at least 8 h, at the sleep laboratory within our department. The overnight sleep polysomnography (SOMNO check 2; Weinmann, Seitingen-Oberflacht, Germany) included electroence-phalography, electrooculography, submental electromyography, electrocardiography, bilateral anterior tibialis electromyography, nasal airflow measurement, oxygen saturation assessment, thoracoabdominal movement monitoring, body position tracking, and snoring. The sleep parameters were manually scored in accordance with the American Academy of Sleep Medicine (AASM) Manual v2.3 2016 (12). Respiratory sleep patterns were examined following the recommendations provided by AASM (13). Apnoea was defined as the cessation of airflow for a minimum duration of 10 s, accompanied by respiratory effort, while hypopnea was characterized by a reduction in airflow of at least 30% lasting for at least 10 s and associated with either a decrease in oxygen saturation of ≥3% or arousal. TS90 referred to the percentage of time spent during sleep with oxygen saturation levels < 90%. The AHI was calculated to assess severity, referred to as the average number of apnoea and hypopnea events per hour. Individuals with an AHI ≥5 event/h were classified as having OSA, whereas those with an AHI of < 5event/h were categorized as primary snorers.

### Positive airway pressure titration

All study participants diagnosed with COPD-OSA underwent an auto-adjusting CPAP titration (SOMNOset, Weinmann GmbH&Co.KG, Hamburg, Germany) in a sleep laboratory setting. We selected the 95th percentile of individual pressure results to describe the effective CPAP pressure for the night. Fixed-pressure bilevel positive airway pressure (BPAP) was employed as an alternative therapy in cases where CPAP proved ineffective. The initial inspiratory positive airway pressure (IPAP) and expiratory positive airway pressure (EPAP) were set at 8cm H_2_O and 4cm H_2_O, respectively. IPAP and/or EPAP was increased by at least 1 cm H_2_O apiece with an interval no shorter than 5 min, until eliminating obstructive respiratory events.

### Positive airway pressure adherence support

Our approach to improving PAP adherence consisted of early intensive education and support, followed by regular and ongoing contact with patients. In the first month after the in-home delivery of the PAP machine, the patient was visited every week; thereafter, the follow-up was carried out via monthly telephone calls for the remainder of the 20-week study period. During in-home visits, staff downloaded pressure, residual AHI, air leak, and adherence data from the PAP machine and reviewed them with the patients. PAP adherence was defined as “excellent” if total cumulative hours of use were ≥ 4 h per night for > 70% of all nights available for use (14).

### Body composition measurements

The human body composition was estimated using a multifrequency bioimpedance analysis (BIA) with the InBody S10 analyser (Biospace Co., Ltd, Seoul, South Korea). The BIA measurements were performed by trained staff following standardized protocols. The BMI, fat-free mass (FFM), fat-free mass index (FFMI), fat mass (FM), and fat mass index (FMI) were determined utilizing the Biospace Inbody S10 composition analyser.

### Cardiopulmonary exercise testing

In the present study, an incremental exercise test was conducted on a MasterScreen CPX electrically braked cycle ergometer (CareFusion Germany 234 GmbH) with a pedalling cadence of approximately 60 revolutions per minute and terminated upon symptom limitation or in the presence of electrocardiographic changes. After a 3-min rest period followed by 3 min of unloaded pedalling, the work rate was progressively increased (ramp) at a rate of 5–10 Watts per min, based on the spirometry results of each subject. Upon cessation of exercise, participants were requested to articulate their primary reason for discontinuing physical activity, such as experiencing breathing difficulties or discomfort in the legs, or any other documented rationale. The intensity of PR was determined through cardiopulmonary exercise testing, specifically targeting a range of 50–70% of peak oxygen uptake, which is defined as moderate-intensity PR exercise. Similar to previous studies, these cutoff values were determined based on the prognostic significance of patients’ maximal oxygen uptake (15).

### PR intervention

The patients underwent conventional exercise training 5 days/week for 40 min, with 10 min of warm-up before training and 10 min of relaxation exercises after training. The exercise component of warm-up incorporated an individualized exercise programme consisting of various types of training, such as walking and functional strength exercises. Moreover, relaxation exercises included stretching and walking. The exercise programme consisted of 20 min of stationary cycling using an upper limb and lower limb coordination exercise machine (Jiangsu Tianrui Medical Equipment Co., Ltd, Nanjing, China), starting at 50% of the maximal load achieved during an exercise test. The load was progressively increased by 10 W if the patient’s heart rate and oxygen saturation were stable and the exercise was well tolerated. Electrocardiogram (ECG) signals and peripheral oxygen saturation (SpO_2_) levels were monitored during the exercise session and within 1 h after the exercise.

To ensure patients’ safety, if the SpO_2_ was < 85%, blood pressure was > 200/100 mmHg (1 mmHg = 0.133 kPa), or the heart rate reached 90% of the maximum value during the cardiopulmonary exercise testing, the exercise was stopped. Furthermore, the physiotherapist reported the case when the patient experienced severe shortness of breath and could not tolerate exercise subjectively. The patients were given a 5-min rest before continuing their training. Herein, we used the criteria for patients who repeatedly refused rehabilitation training more than 3 times among those who cooperated poorly and were withdrawn from the study.

### Sample size

The sample size was calculated as a function of the expected change in the results of the 6MWD. To study the efficacy of pulmonary rehabilitation on patients with COPD-OSA overlap syndrome who utilize positive airway pressure therapy, 6-metre walking distance increased by 25 m was considered clinically significant. In order to detect a 1standard deviation difference between the arms, at least 15 patients were required in each group under the assumption of a 2-sided type I error of 5% and a power of 90% (t-test). An expected loss to follow-up of 5% (in terms of missing primary outcome data) implied that a sample size of 16 patients was required. Because of low positive airway pressure adherence (16, 17), the trial was ended after the sample size reached >90% of the estimation.

### Statistical analysis

The data were analysed using SPSS 22.0 software (IBM Corp, Armonk, NY, USA). Prior to statistical analysis, the Kolmogorov–Smirnov and Shapiro–Wilk tests assessed the normality of continuous data. The continuous and categorical variables were presented by mean, standard deviation, absolute, and relative frequencies. Both χ^2^ tests and t-tests were used to evaluate the baseline differences between the groups. Non-normally distributed variables were compared by using the Mann–Whitney *U* test. For all analyses, *p* < 0.05 (2-tailed) was considered statistically significant.

## RESULTS

**Fig. 1:**
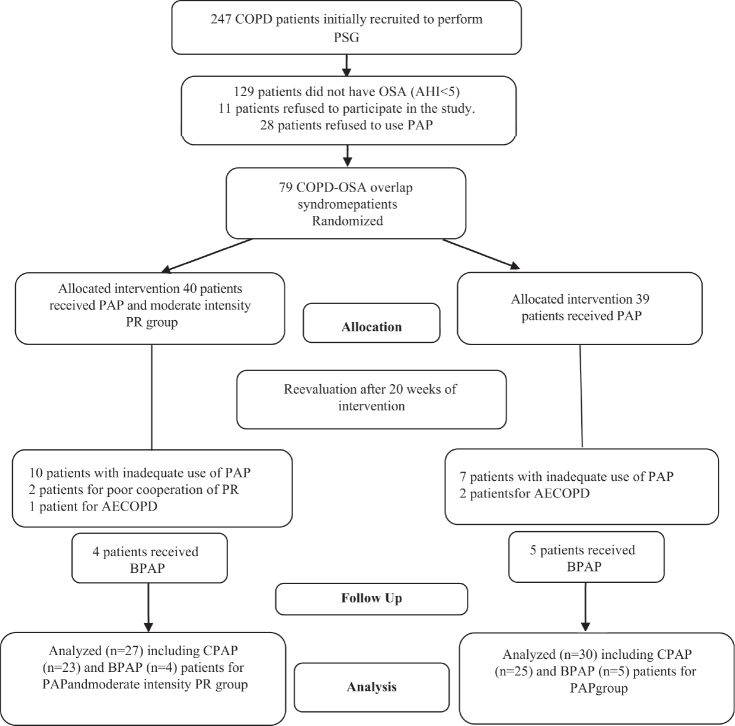
Flowchart showing the patient selection process leading to the final cohort. PAP: positive airway pressure; PR: pulmonary rehabilitation.

During the study period, 247 patients with COPD snoring at night underwent 1 night (≥8 h) of PSG study. In total, 79 patients (62.9 ± 7.2 years, 51.9% males), who fulfilled the inclusion criteria were randomized single-blind into the intervention group (IG) (*n* = 40) or the control group (CG) (*n* = 39) using a computerized list. Subsequently, 22 patients were lost to follow-up, including 17 patients with inadequate use of PAP, 3 patients due to acute exacerbation of COPD, and 2 patients due to poor cooperation during PR. The final cohort comprised 57 patients at the end of the 20^th^ week (Fig. 1). All baseline characteristics of the 2 groups are presented in Tables I and II.

**Table I:** Baseline characteristics of patients with COPD-OSA overlap syndrome

Characteristics	IG (*n* = 40)	CG (*n* = 39)	*p*-value
Age, years, mean (SD)	62.0 (7.1)	62.7 (7.5)	0.640
Gender (male/female)	25/15	21/18	0.436
Height, m, mean (SD)	1.7 (0.1)	1.7 (0.1)	0.984
Weight, kg, mean (SD)	72.5 (13.4)	74.2 (13.5)	0.585
Body mass index, kg/m^2^, mean (SD)	24.7 (3.0)	25.5 (4.6)	0.381
Underlying disease, *n* (%)			
Coronary disease	13 (32.5%)	14 (35.9%)	0.750
Diabetes	12 (30.0%)	11 (28.2%)	0.861
Hypertension	25 (62.5%)	20 (51.3%)	0.314
Smoking status, *n* (%)			0.892
Current smoker	25 (62.5%)	26 (66.7%)	
Ex-smoker	10 (25.0%)	8 (20.5%)	
Non-smoker	5 (12.5%)	5 (12.8%)	
GOLD stage, *n* (%)			0.351
2	12 (30.0%)	8 (20.5%)	
3	25 (62.5%)	29 (74.4%)	
4	3 (7.5%)	2 (5.1%)	
Pulmonary function, mean (SD)			
FEV_1_ (%predicted)	44.6 (8.4)	44.9 (8.3)	0.896
FEV_1_ (L)	1.1 (0.4)	1.1 (0.3)	0.854
FEV_1_/FVC	0.5 (0.1)	0.5 (0.1)	0.789
OSA severity, *n* (%)			0.782
Mild OSA (5 ≤ AHI< 15)	14 (35.0%)	11 (28.2%)	
Moderate OSA (15 ≤ AHI < 30)	14 (35.0%)	14 (35.9%)	
Severe OSA (AHI ≥ 30)	12 (30.0%)	14 (35.9%)	
AHI, event/h, mean (SD)	20.0 (10.8)	20.6 (9.0)	0.776
Medication, *n* (%)			
Beta-2 agonist use, *n* (%)	24 (60.0%)	19 (48.7%)	0.314
Anticholinergic use, *n* (%)	22 (55.0%)	19 (48.7%)	0.576
Inhaled corticosteroid use, *n* (%)	16 (40.0%)	15 (38.5%)	0.889

Student’s t-test or χ^2^ test.

IG: intervention group; CG: control group; FEV_1_: forced expiratory volume in 1 s; FVC: forced vital capacity; AHI: apnoea–hypopnea index.

**Table II:** Outcomes in intervention group and control group

Variables	IG (*n* = 27)	CG (*n* = 30)	*p*_2_-value
*Physical endurance*			
6MWD, m, mean (SD)			
Baseline	269.5 (48.9)	247.7 (61.7)	0.148
Week 20	317.3 (58.7)	241.3 (37.9)	< 0.001
* p*_1_ value	0.003	0.599	
*Body composition*			
Weight, kg, mean (SD)			
Baseline	72.8 (12.3)	74.7 (13.5)	0.577
Week 20	67.8 (11.1)	77.1 (12.4)	0.004
*p*_1_ value	< 0.001	0.001	
Body mass index, kg/m^2^, mean (SD)			
Baseline	24.7 (2.8)	25.7 (4.6)	0.317
Week 20	22.3 (2.2)	26.5 (4.2)	< 0.001
*p*_1_ value	< 0.001	0.001	
Fat mass, kg, mean (SD)			
Baseline	24.9 (9.1)	27.1 (9.1)	0.369
Week 20	20.6 (8.6)	30.3 (8.9)	< 0.001
*p*_1_ value	< 0.001	< 0.001	
Fat mass ndex, kg/m^2^, mean (SD)			
Baseline	8.4 (2.9)	9.3 (3.3)	0.253
Week 20	6.9 (2.7)	10.5 (3.3)	< 0.001
*p*_1_ value	< 0.001	< 0.001	
Fat-free mass, kg, mean (SD)			
Baseline	47.9 (5.5)	47.7 (5.8)	0.863
Week 20	47.2 (5.4)	46.8 (5.0)	0.754
*p*_1_ value	0.070	0.115	
Fat-free mass index,kg/m^2^, mean (SD)			
Baseline	16.3 (0.7)	16.4 (1.5)	0.795
Week 20	16.0 (0.8)	16.0 (1.0)	0.984
*P*_1_ value	0.100	0.104	
*Respiratory function*			
FEV_1_% predicted, mean (SD)			
Baseline	43.7 (9.7)	44.0 (8.2)	0.890
Week 20	43.6 (9.8)	44.1 (7.2)	0.824
*p*_1_ value	0.722	0.822	
FEV_1_, L, mean (SD)			
Baseline	1.1 (0.3)	1.1 (0.3)	0.866
Week 20	1.1 (0.3)	1.1 (0.3)	0.983
*p*_1_ value	0.285	0.961	
Forced expiratory volume in 1 s/Forced vital capacity, mean (SD)			
Baseline	0.5 (0.1)	0.5 (0.1)	0.873
Week 20	0.5 (0.1)	0.5 (0.1)	0.308
*p*_1_ value	0.073	0.855	
mMRC, mean (SD)			
Baseline	2.2 (0.7)	2.3 (0.7)	0.513
Week 20	1.0 (0.9)	1.5 (0.6)	0.005
*p*_1_ value	< 0.001	< 0.001	
*Polygraphic nocturnal parameters*			
Adherence, h/night, mean (SD)	4.7 (0.6)	4.8 (0.6)	0.698
CPAP pressure, cm H_2_O, mean (SD)	*n* = 23, 8.3 (1.7)	*n* = 25, 7.6 (1.7)	0.191
Apnoea–hypopnea index, event/h, mean (SD)			
Baseline	21.3 (11.4)	21.8 (9.4)	0.867
Week 20	3.6 (1.1)	4.4 (1.5)	0.040
*p*_1_ value	< 0.001	< 0.001	
Night SpO_2_ mean, %, mean (SD)			
Baseline	88.1 (1.3)	88.4 (2.0)	0.473
Week 20	92.1 (1.0)	90.2 (2.1)	< 0.001
*p*_1_ value	< 0.001	< 0.001	
L-SaO_2_, %, mean (SD)			
Baseline	72.9 (6.7)	70.9 (6.8)	0.278
Week 20	87.7 (2.9)	84.7 (5.0)	0.008
*p*_1_ value	< 0.001	< 0.001	
TS90, %, mean (SD)			
Baseline	10.0 (6.8)	8.3 (7.1)	0.360
Week 20	3.6 (1.6)	4.7 (1.8)	0.013
*p*_1_ value	< 0.001	0.009	
*Arterial blood gasses*			
Potential of hydrogen, mean (SD)			
Baseline	7.3 (0.1)	7.3 (0.1)	0.718
Week 20	7.4 (0.0)	7.3 (0.0)	< 0.001
*p*_1_ value	< 0.001	0.004	
PaO_2_, mmHg, mean (SD)			
Baseline	57.6 (8.5)	61.3 (9.7)	0.132
Week 20	68.0 (5.2)	63.0 (5.5)	0.001
*p*_1_ value	< 0.001	0.404	
PaCO_2_, mmHg, mean (SD)			
Baseline	50.6 (6.3)	50.0 (7.7)	0.751
Week 20	45.9 (5.7)	46.7 (5.1)	0.570
*p*_1_ value	0.014	0.055	
*Basal activity of daily living*			
Barthel Index, mean (SD)			
Baseline	67.2 (12.0)	63.2 (9.2)	0.157
Week 20	77.2 (11.6)	68.5 (11.2)	0.006
*p*_1_ value	< 0.001	< 0.001	

*p*_1_, Student’s t-test of paired-samples was used for comparisons to entry (baseline); *p*_2_*,* Student’s t-test of independent samples was used for comparisons between intervention group (IG) and control group (CG).

L-SaO_2_(%): lowest nocturnal oxygen saturation; TS90%: percentage of total sleep time with oxygen saturation < 90%; mMRC: modified Medical Research Council; PaO_2_: arterial oxygen partial pressure; PaCO_2_: arterial carbon dioxide partial pressure.

### Primary endpoints

For physical endurance, there were marked improvements in the IG compared with the baseline and were significantly higher than CG at 20 weeks (all *p* < 0.01). However, no statistically significant differences were detected on 6MWT in the CG (*p* > 0.05) (Table II).

### Secondary endpoints

The adherence of PAP and positive pressure did not differ significantly between the 2 groups (all *p* > 0.05). Compared with the baseline, obvious improvements were noted in terms of AHI and other oxygen deprivation parameters in the 2 groups (all *p* < 0.01). Moreover, the IG demonstrated a significantly lower AHI and TS90% compared with the CG, along with markedly higher L-SaO_2_% and night SpO_2_ % (all *p* < 0.05) (Table II).

At 20 weeks, the 2 groups exhibited obvious improvements in mMRC compared with the baseline (all *p* < 0.001), and the IG had a significantly lower mMRC compared with the CG (*p* = 0.005). However, no statistically significant difference was observed in FEV_1_ (%predicted), FEV_1_,and FEV_1_/FVC in the 2 groups (*p* > 0.05) (Table II).

For body composition, the IG had a significantly lower weight, BMI, FM, and FMI compared with the baseline and CG (all *p* < 0.01), while the CG had markedly higher weight, BMI, FM, and FMI compared with the baseline (all *p* < 0.01) (Table II).

For arterial blood gasses, statistically obvious improvements were observed in IG compared with those at the baseline (all *p* < 0.05). The IG had significantly higher PaO_2_ compared with the CG (*p* < 0.01), but no difference was detected in PaCO_2_ (*p* > 0.05) (Table II).

For the basal activity of daily living, both the IG and CG exhibited significant improvements in BI compared with the baseline. However, the IG demonstrated a more pronounced improvement compared with the CG (all *p* < 0.01) (Table II).

## DISCUSSION

In this randomized clinical single-blind trial, we evaluated the efficacy of pulmonary rehabilitation combining aerobic exercise with PAP, compared with PAP alone in patients with COPD-OSA overlap syndrome. IG showed significantly high physical endurance within 20 weeks after PR. These results indicated an obvious improvement in body composition and intermittent hypoxia during sleep. In addition, the motor ability improved dramatically due to the addition of moderate intensity PR. However, the outcomes did not demonstrate any significant effect on pulmonary function.

Because fatigue was more prominent in patients with COPD-OSA overlap syndrome than in sleep apnoeic patients and did not ameliorate after treatment with continuous positive airways pressure (CPAP), their daily life activity decreased (18). A minimum level of muscle strength and endurance is necessary to maintain the ability to perform the activities of daily living and for functional independence in old age and/or in the presence of disease-related limitations (19). The current study showed that the 6-metre walking distance of patients before rehabilitation intervention was only 258±57 m, which corresponds with the studies by Limsuwat et al. (20) and Vitacca et al. (7), wherein the overlap syndrome patients who participated in exercise-based PR were likely to have a moderate or high physical endurance level. Schucher et al. (21) demonstrated that the PaCO_2_ recruitment threshold can be normalized by intermittent nasal positive pressure ventilation as well as the PaCO_2_ under spontaneous breathing. The load of the respiratory pump decreases due to an increase of the inspiratory muscle strength. Soler et al. (10) proposed that PR programmes should include sleep assessment in patients with moderate to severe COPD and interventions that could reduce the impact of OSA in COPD.

The COPD-OSA overlap syndrome patients had significantly lower AHI and TS90%, as well as markedly higher L-SaO_2_% and mean night SpO_2_% due to PAP therapy. Although their intermittent hypoxia during sleep improved, CG patients expressed significantly more physical inactivity than IG patients; this phenomenon persisted even after 20 weeks of PAP therapy. Our study indicates that a sole application of PAP does not improve patients’ physical activities and effort tolerance, but combined with aerobic exercise effectively enhances their exercise endurance. This phenomenon may be attributed to the fact that aerobic exercise can enhance cardiovascular function, optimize oxygen utilization, improve muscle performance, and consequently augment athletic endurance. On the other hand, patients who participated in additional aerobic exercise spent more time in physical endurance and chose different physical activities compared with CG patients.

Schreiber et al. (22) found a significant improvement in arterial blood gases after 1 year of CPAP. But Dumitrache-Rujinski et al. (23) showed a different result. They found patients whose COPD exacerbations with respiratory acidosis are difficult to manage, especially when OSA and obesity are associated, despite using non-invasive ventilation associated with oxygen therapy. Our study also demonstrated a significant improvement in arterial blood gases among all patients with COPD-OSA overlap syndrome following treatment with PAP, which may be attributed to the absence of obesity in our enrolled patient cohort. In the IG patients, acidosis was corrected, PaO_2_ was significantly increased, and hypoxia was improved, while PaCO_2_ decreased significantly. Although CG improved, it did not reach statistical significance. Further investigation is warranted to determine whether the weight loss attributed to PR confers additional benefits.

The effect of PAP on bodyweight is as yet controversial. Herein, the body composition was evaluated by multifrequency bioimpedance analysis. The BMI, FM, and FMI of patients in the CG showed an increasing trend, similar to Feng et al. (24). Choi et al. (25) described that being overweight may protect moderate-to-severe OSA patients from the risk of COPD (i.e., overlap syndrome), although BMI is a well-known risk factor for OSA. Our study showed a significant decrease in bodyweight, BMI, FM, and FMI due to the 20 weeks of aerobic exercise in IG. OSA is commonly associated with obesity and can be improved by weight loss, related to changes in the upper airway size that could improve the condition (25). Schreiber et al. (26) also showed that BMI values are commonly considered cutoff values for the prediction of OSA in the general population and may not be accurate in a subgroup of patients with COPD.

Previous studies have heterogeneous data on lung function changes after CPAP treatment in the overlap syndrome. Toraldo et al. (2) described an increase in FEV_1_ of 15% (*p* < 0.0015) at 24 months compared with the baseline values. However, similar to Mansfield et al. (27), we did not find any differences in the pulmonary function tests after 20 weeks of PAP treatment in the whole patient population; although their subjective dyspnoea symptoms improved, mMRC decreased significantly. Schreiber et al. (22) explained that a population of overlap patients treated with CPAP might experience a different change in airflow obstruction after 1 year depending on the severity of baseline obstruction; nonetheless, additional studies are required for longer observation periods.

PR is beneficial to patients with COPD (28), and generally improves physical performance, activities of daily living, and dyspnoea (29), and the health-related quality of life and the BODE index have been improved in the previously mentioned studies (30, 31). Our previous studies also confirmed that PR improved exercise capacity in COPD patients (32, 33), though PSG was not tested for sleep in those patients. Poor sleep quality as a component of this multimorbidity spectrum has a negative impact on COPD-OSA overlap syndrome, and another study assessed the correlation based on nocturnal sleep parameters (34). The coexistence of COPD and OSA can cause multiple systemic damage, and the main physiological mechanisms are continuous hypoxia and intermittent hypoxia (35). Soler et al. (10) recommended that pulmonary rehabilitation programmes consider including a sleep assessment in patients with moderate to severe COPD and interventions that help reduce the impact of OSA in COPD.

In this study, OSA was screened from COPD patients, and the comorbidity rate was also high at 47.77%. Thus, the current results showed that after 20 weeks of PAP there are significant improvements in terms of AHI and other oxygen deprivation parameters within the 2 groups. At the same time, IG showed a markedly large improvement. These results demonstrated that initiation of PAP therapy and moderate pulmonary rehabilitation is associated with improved prognosis in patients with overlap syndrome. Ventilation with CPAP is the gold-standard therapy for OSA. However, a recent study suggested that a novel mode of ventilation, Bilevel-auto, could be equally effective in treating patients unable to tolerate CPAP (36), 9 patients in our study received BPAP as they were intolerant to CPAP.

### Limittions

Nevertheless, the present study has several limitations: (1) this was a two-centre study, and the results may not generally be applicable to all patients with COPD-OSA overlap syndrome; (2) a significant number of dropouts could be attributed to inadequate use of PAP and missing data cases that complicated the statistical analysis and limited the study results; (3) the study was unblinded for patients and staff, although during the evaluation of physical tests all researchers were blinded to the allocation group; (4) this study did not give resistance training, and the body composition did not show any change in FFM and FFMI.

### Conclusions

The addition of moderate intensity pulmonary rehabilitation for 20 weeks could increase the level of physical endurance and motor abilities, and in turn improve sleep apnoea, nocturnal hypoxia, body composition, and arterial blood gases in patients with COPD-OSA overlap syndrome who use positive airway pressure.

## Data Availability

The data that support the findings of this study are available on request from the corresponding author.
